# A possible role of microglia-derived nitric oxide by lipopolysaccharide in activation of astroglial pentose-phosphate pathway via the Keap1/Nrf2 system

**DOI:** 10.1186/s12974-016-0564-0

**Published:** 2016-05-04

**Authors:** Takuya Iizumi, Shinichi Takahashi, Kyoko Mashima, Kazushi Minami, Yoshikane Izawa, Takato Abe, Takako Hishiki, Makoto Suematsu, Mayumi Kajimura, Norihiro Suzuki

**Affiliations:** Department of Neurology, Keio University School of Medicine, 35 Shinanomachi, Shinjuku-ku, 160-8582 Tokyo, Japan; Department of Neurology, Osaka City University Graduate School of Medicine, Osaka-shi, 545-8585 Osaka , Japan; Department of Biochemistry, Keio University School of Medicine, Shinjuku-ku, 160-8582 Tokyo, Japan; Clinical and Translational Research Center, Keio University School of Medicine, Shinjuku-ku, 160-8582 Tokyo, Japan; JST Exploratory Research for Advanced Technology (ERATO) Suematsu Gas Biology Project, Shinjuku-ku, 160-8582 Tokyo , Japan

**Keywords:** Ara-C (cytosine β-d-arabinofuranoside hydrochloride), Astrocyte, LME (l-leucine methyl ester), RNS (reactive nitrogen species), ROS (reactive oxygen species), TLR4 (Toll-like receptor 4)

## Abstract

**Background:**

Toll-like receptor 4 (TLR4) plays a pivotal role in the pathophysiology of stroke-induced inflammation. Both astroglia and microglia express TLR4, and endogenous ligands produced in the ischemic brain induce inflammatory responses. Reactive oxygen species (ROS), nitric oxide (NO), and inflammatory cytokines produced by TLR4 activation play harmful roles in neuronal damage after stroke. Although astroglia exhibit pro-inflammatory responses upon TLR4 stimulation by lipopolysaccharide (LPS), they may also play cytoprotective roles via the activation of the pentose phosphate pathway (PPP), reducing oxidative stress by glutathione peroxidase. We investigated the mechanisms by which astroglia reduce oxidative stress via the activation of PPP, using TLR4 stimulation and hypoxia in concert with microglia.

**Methods:**

In vitro experiments were performed using cells prepared from Sprague–Dawley rats. Coexisting microglia in the astroglial culture were chemically eliminated using l-leucine methyl ester (LME). Cells were exposed to LPS (0.01 μg/mL) or hypoxia (1 % O_2_) for 12–15 h. PPP activity was measured using [1-^14^C]glucose and [6-^14^C]glucose. ROS and NO production were measured using 2′,7′-dichlorodihydrofluorescein diacetate and diaminofluorescein-FM diacetate, respectively. The involvement of nuclear factor-erythroid-2-related factor 2 (Nrf2), a cardinal transcriptional factor under stress conditions that regulates glucose 6-phosphate dehydrogenase, the rate-limiting enzyme of PPP, was evaluated using immunohistochemistry.

**Results:**

Cultured astroglia exposed to LPS elicited 20 % increases in PPP flux, and these actions of astroglia appeared to involve Nrf2. However, the chemical depletion of coexisting microglia eliminated both increases in PPP and astroglial nuclear translocation of Nrf2. LPS induced ROS and NO production in the astroglial culture containing microglia but not in the microglia-depleted astroglial culture. LPS enhanced astroglial ROS production after glutathione depletion. U0126, an upstream inhibitor of mitogen-activated protein kinase, eliminated LPS-induced NO production, whereas ROS production was unaffected. U0126 also eliminated LPS-induced PPP activation in astroglial–microglial culture, indicating that microglia-derived NO mediated astroglial PPP activation. Hypoxia induced astroglial PPP activation independent of the microglia–NO pathway. Elimination of ROS and NO production by sulforaphane, a natural Nrf2 activator, confirmed the astroglial protective mechanism.

**Conclusions:**

Astroglia in concert with microglia may play a cytoprotective role for countering oxidative stress in stroke.

**Electronic supplementary material:**

The online version of this article (doi:10.1186/s12974-016-0564-0) contains supplementary material, which is available to authorized users.

## Background

Although multiple mechanisms underlie the pathophysiology of ischemic stroke, there is increasing evidence that inflammation accounts for its progression [1, 2]. A robust inflammatory reaction characterized by peripheral leukocyte influx into the cerebral parenchyma and the activation of endogenous microglia follows focal cerebral ischemia [3–7]. Reactive oxygen species (ROS) and reactive nitrogen species (RNS) play pivotal roles in neuronal damage. ROS and/or RNS are, at least in part, produced by cerebral parenchymal cells, including neurons and glial cells, as well as peripheral inflammatory cells such as leukocytes. The origin of ROS appears to be from activated NADPH oxidase (NOX), and ROS, in turn, reacts with nitric oxide (NO) produced by activated inducible nitric oxide synthase (iNOS), leading to peroxynitrite generation, which is a highly toxic reactive species that can induce lipid peroxidation and protein oxidative damage [8]. Thus, the intrinsic protective mechanism that eliminates ROS may play an important role in countering post-stroke inflammation.

Toll-like receptors (TLRs) mediate innate immune response, and recent findings suggest that they also mediate post-stroke inflammatory responses [9]. There are at least 11 TLR proteins in mammals [10], and TLR4 is implicated in the pathogenesis of stroke-related inflammation. TLR4 is predominantly expressed in both microglia and astroglia, and also in neurons [11]. Although lipopolysaccharide (LPS), a component of bacterial cell walls, is a well-known classical ligand of TLR4, extracellular matrix and dysfunctional mitochondria released from damaged cells have been reported to be endogenous ligands of TLR4 [12]. TLR4 stimulation of microglia plays a harmful role in stroke through downstream signaling pathways: myeloid differentiation protein 88–nuclear factor κB (NFκB), mitogen-activated protein kinase (MAPK), also called extracellular signal-regulated kinase 1/2, and Janus kinases, which are signal transducers and activators of transcription. In particular, the activation of NFκB induces NOX and iNOS, which enhance ROS and NO production by microglia.

Astroglia express TLR4 even in the human brain, and the stimulation of TLR4 by LPS in cultured astroglia induces pro-inflammatory responses [13]. In contrast, LPS may also induce an astroglial anti-inflammatory, protective function via the pentose phosphate pathway (PPP) [14]. PPP is a shunt pathway of glycolysis and produces NADPH, which recycles the oxidized from of glutathione (GSH) back to the reduced form, leading to the removal of ROS by GSH peroxidase. Thus, PPP activity is an index of endogenous neuroprotective function. In fact, astroglial PPP activity is much higher than that of neurons [15], and PPP activation plays an important role in countering oxygen stress. The activation of astroglial TLR4 by LPS activates astroglial PPP through NO [14, 16, 17]. The exact mechanism by which NO activates PPP remains to be elucidated.

The rate-limiting enzyme of PPP, glucose 6-phosphate dehydrogenase (G6PDH), is regulated both allosterically and transcriptionally. Under stress conditions, the master regulator of the anti-stress response, the Kelch-like ECH-associated protein 1 (Keap1)/nuclear factor-erythroid-2-related factor 2 (Nrf2) system induces various enzymes via transcriptional regulation mediated by Nrf2. In fact, G6PDH also harbors an antioxidant response element (ARE) to which Nrf2 binds and initiates the transcription of G6PDH, leading to PPP activation [18]. Nrf2 is bound to its adaptor protein Keap1 and is constantly degraded by the ubiquitin–proteasome system in the cytosol. However, several signals can affect the cysteine residues of Keap1 to induce a conformational change for releasing Nrf2. Free Nrf2 translocates into the nucleus and initiates transcription. Numerous reports show that both ROS and/or NO can induce Keap1 conformational change and Nrf2 activation [19–22]. Thus, TLR4-mediated production of ROS, NO, or both by astroglia is a potential mediator.

Another important issue that should be clarified is the cellular origin of ROS or NO. There is no doubt that LPS induces ROS and NO production in microglia. However, whether astroglial ROS and NO production are induced by LPS remains to be established. Most studies describing the astroglial response to LPS have been based on cell culture experiments [23]. Usually, an astroglial culture contains a small (<10 %) population of microglia. If LPS acts on microglia, even on <10 % of the total cells, microglia can produce sufficient ROS and NO in response to LPS.

The present study used a conventional secondary astroglial culture prepared from pups of Sprague–Dawley rats. To eliminate microglial cells, the conventional astroglial culture was treated with cytosine β-d-arabinofuranoside hydrochloride (Ara-C), followed by l-leucine methyl ester (LME) [24, 25]. This procedure completely eliminates Iba1-positive cells. We used both microglia-present and microglia-absent astroglial cultures and evaluated PPP activity, as well as ROS and NO production in response to LPS.

## Methods

### Animals

Timed-pregnant Sprague–Dawley rats were obtained from Japan SLC, Inc. (Hamamatsu, Japan). Male C57BL/6 (wild-type (WT)) mice (Japan SLC, Inc.) and Nrf2 gene knockout (KO) mice (on C57BL/6 background, originally generated by Dr. Masayuki Yamamoto) were used in this study. Nrf2-KO mice were genotyped by PCR amplification of genomic DNA extracted from tail snips. All animal procedures were performed in accordance with The Animal Experimentation Guidelines of Keio University School of Medicine and were approved by the Committee on Animal Care and Use, Keio University (08097-[15]).

### Chemicals

Chemicals and materials were obtained from the following sources: Insta-Fluor Plus and Hyamine hydroxide 10-X from Perkin-Elmer Life Sciences (Boston, MA, USA); d-[1-^14^C]glucose (specific activity, 2.035 GBq/mmol) and d-[6-^14^C]glucose (specific activity, 2.035 GBq/mmol) from American Radiolabeled Chemicals, Inc. (St. Louis, MO, USA); normal goat serum from Jackson ImmunoResearch (West Grove, PA, USA); rabbit Iba1 antibody from Wako Pure Chemical Industries (Osaka, Japan); mouse monoclonal anti-glial fibrillary acidic protein (GFAP) from Sigma (St. Louis, MO, USA); goat polyclonal antibody against TLR4 from Santa Cruz Biochemistry (Delaware, CA, USA); 1,4-diamino-2,3-dicyano-1,4-bis[2-aminophenylthio] butadiene (U0126), a selective inhibitor of MAPK/ERK kinase1/2 (MEK1/2) from Cell Signaling Technology (Danvers, MA, USA); mouse monoclonal antibody against anti-β actin from Abcam (Cambridge, UK); rabbit anti-Nrf2 (H-300) polyclonal antibody, rhodamine-conjugated goat anti-mouse (for GFAP), anti-rabbit (for Nrf2) IgG antibody, and fluorescein-isothiocyanate (FITC)-conjugated goat anti-rabbit (for Iba1) antibody from Santa Cruz Biochemistry; Dulbecco’s modified Eagle media (DMEM) with or without glucose, penicillin, and streptomycin from Life Technologies (Grand Island, NY, USA); defined fetal bovine from HyClone Laboratories (Logan, UT, USA); 2′,7′-dichlorodihydrofluorescein diacetate (H_2_DCFDA) and monochlorobimane (MCB) from Molecular Probe Inc. (Eugene, OR, USA); diaminofluorescein-FM diacetate (DAF-FM DA) from Sekisui Medical (Chuo-ku, Tokyo, Japan); and all other chemicals were obtained from Sigma (St. Louis, MO, USA).

### Preparation of cells

Primary astroglial cultures were prepared from the rat or mouse cerebral cortices 24–48 h after birth [26]. Dissociated cells from the cerebral cortices (2.5 × 10^5^ cells/mL for rat and 5.0 × 10^5^ cells/mL for mouse) were plated (15 mL/flask) in uncoated 75-cm^2^ culture flasks (Sumitomo Bakelite, Tokyo, Japan) and cultured in a glucose-containing medium (final concentration, 12 mmol/L of d-glucose) comprising DMEM with 10 % (*v*/*v*) fetal bovine serum, penicillin (100 U/mL), and streptomycin (100 μg/mL) at 37 °C in humidified air containing 21 % O_2_ and 7 % CO_2_ (day 0). The culture medium was changed every 2 days until the cultures reached confluence. On day 11, the adherent cells were treated with trypsin–EDTA solution, suspended in a fresh high (12 mmol/L)-glucose medium, and placed in uncoated 12-well culture plates (0.8 mL/well, respectively; Nalge Nunc, Rochester, NY, USA) or in 25-cm^2^ culture flasks (5 mL/flask; Nalge Nunc). For immunohistochemical staining, the cells were plated (2 mL/dish) on 35-mm glass-bottomed dishes (Matsunami Glass, Industry, Osaka, Japan) precoated with poly-l-lysine. Some cells were grown on glass-bottomed 24-well culture plates (EZView culture plate LB, Iwaki, Tokyo, Japan) for fluorometric assay. From the day after subculturing, the cells were cultured in DMEM, fetal bovine serum, penicillin, and streptomycin and a final d-glucose concentration of 12 mmol/L for 10 days. The culture medium was changed twice a week, and the cells were used once they reached confluence (on day 21 in vitro). Some secondary astroglial cells were treated with Ara-C followed by LME to eliminate microglia.

Primary neuronal cultures were prepared from the striatum of fetal rats on embryonic day 16, as previously described [26]. Mechanically dissociated cells were placed in 12-well culture plates (0.8 mL/well, respectively) or 25-cm^2^ culture flasks (5 mL/flask) coated with poly-l-lysine (5 μg/mL). For the neuronal cultures, viable cells (1.5 × 10^6^ cells/mL) that excluded trypan blue were placed in the cultures, and Ara-C (10 μmol/L) was added 72 h later to induce mitotic arrest of the astroglia. The cells were cultured in a glucose medium (final concentration, 12 mmol/L d-glucose) at 37 °C in humidified air containing 21 % O_2_ and 7 % CO_2_. In vitro assays were performed using cultures that were 7 or 8 days old. The nutrient medium remained untouched until the experiments were initiated.

Microglial cells were obtained by collecting floating or loosely adherent cells in primary astroglial culture and were placed in 12-well culture plates or glass-bottomed dishes precoated with poly-l-lysine [27].

### Depletion of microglia from astroglial cultures

Usually, secondary astroglial cultures contain only small amounts (<10 % of total cells) of microglia, as previously described [27, 28]. To eliminate all possible metabolic effects of microglial contamination, the treatment with a combination of Ara-C and LME was applied according to the method described by Pont-Lezica et al. [25]. Briefly, 10 μmol/L (final concentration in nutrient medium) of Ara-C was added to the astroglial culture 3 days prior to the assay. After 48 h, cells were further incubated with 50 mmol/L of LME (pH adjusted to ~7.4) for 60 min. Cells were then washed with phosphate-buffered saline (PBS) without Ca^2+^ and Mg^2+^ and further cultured with a regular nutrient medium for 24 h. This treatment destroyed microglia by osmotic effects and yielded almost 100 % pure astroglial cell culture. The exact mechanism by which LME eliminates microglia remains to be elucidated. It has been speculated that LME is internalized by microglia, wherein it causes lysosomal disruption and subsequent apoptosis. In fact, macrophages and microglia are characteristically rich in lysosomes, causing them to be particularly vulnerable upon exposure to LME treatment [29, 30].

### Experimental protocol

To assess the effects of LPS on astroglial PPP activation, ROS/NO production, and immunohistochemistry, the nutrient medium (12 mmol/L) was removed and fresh nutrient medium containing LPS (0.01, 0.1, or 1 μg/mL) was added and kept in a CO_2_ incubator for 15 h. For neuronal cultures, a medium change per se damages cells via glutamate toxicity [31], LPS was directly added to the culture media of cells to obtain final concentrations of 0.01, 0.1, or 1 μg/mL, as described above. When astroglial cells were exposed to 1-isothiocyanato-(4R,S)-(methylsulfinyl)butane (sulforaphane), an Nrf2 activator [32–34] or S-nitroso-*N*-acetyl-dl-penicillamine (SNAP), an NO donor [35], the nutrient medium was replaced with a fresh medium containing the drug or vehicle (dimethyl sulfoxide (DMSO)), and cells were incubated for 15 h. When the effects of sulforaphane (10 μmol/L), U0126 (10 μmol/L), l-buthionine-sulfoximine (BSO; 50 μmol/L)—an inhibitor of GSH synthesis [36]—on LPS-induced ROS/NO production were assessed, cells were pre-incubated with these drugs for 12 h before LPS treatment and further incubated for 12 h with LPS, followed by the measurement of ROS and NO.

We next assessed the effects of hypoxia because it is also well recognized that hypoxic conditions per se shift the cellular metabolism from oxidative to glycolytic in various cells. However, whether hypoxia enhances PPP flux has not been addressed. An early study revealed that chronic hypoxia induced PPP activity in C6 glioma cells [37], but the mechanism that conveys the hypoxic signal for PPP activation is undetermined. In a recent study [38], hypoxia and reperfusion induced PPP activation in astroglia. Although the underlying mechanism was not described, the reperfusion-induced massive production of ROS in association with NO might exert a strong influence on PPP activation. Moreover, the effect of LPS or LPS in combination with hypoxia on astroglial PPP activity has not been reported. In the present study, we examined the effect of LPS stimulation in combination with hypoxia on PPP flux in astroglia. To assess the effect of hypoxia on PPP activation, we placed cells for 12 or 24 h in a hypoxic chamber (multi-gas incubator APM-30DR, ASTEC, Fukuoka-ken, Japan) at 37 °C in humidified air containing 1 % O_2_ and 7 % CO_2_. We assessed the combined effects of LPS (0.01 μg/mL) and hypoxia (1 % O_2_) for 15 h in some neuronal and astroglial cells.

### Measurement of the rates of d-[1-^14^C]glucose and d-[6-^14^C]glucose oxidation to ^14^CO_2_

The rate of [^14^C]glucose oxidation to ^14^CO_2_ was measured using a modification of a previously described method [39] after cells cultured in 25-cm^2^ culture flasks were washed twice with PBS containing no glucose. The assay solutions consisted of Dulbecco’s balanced salt solution (DBSS) containing (mmol/L) 110 NaCl, 5.4 KCl, 1.8 CaCl_2_, 0.8 MgSO_4_, 0.9 NaH_2_PO_4_, and 44 NaHCO_3_, in addition to 2 mmol/L of d-glucose labeled with 1.0 μL/mL of d-[1-^14^C]glucose or d-[6-^14^C]glucose (original concentrations, 3.7 MBq/mL), were added, and the flasks were capped with rubber stoppers containing a center well and incubated at 37 °C for 60 min. The ^14^CO_2_ produced was trapped by a cotton ball placed in the center well containing 100 μL of Hyamine hydroxide 10-X. The reactions were terminated by the injection of 250 μL of perchloric acid (2 mol/L) through the rubber stopper, and the flasks were kept at 4 °C overnight to trap the ^14^CO_2_. The center wells were then transferred to 20-mL glass scintillation counter vials, and 500 μL of ethanol and 10 mL of Insta-Fluor Plus were added. The ^14^C contents of the vials were evaluated in a liquid scintillation counter. The assay solutions consisted of 2.5 mL of DBSS containing 2 mmol/L d-glucose; these solutions were labeled by the addition of 1.0 μL/mL of d-[1-^14^C]glucose or d-[6-^14^C]glucose (original concentrations, 3.7 MBq/mL).

Waniewski and Martin [40] reported that substantial ^14^C counts were obtained from a flask without cells. Accordingly, the ^14^C counts obtained from a flask without cells in which the reaction had been stopped at 60 min were treated as background values and were subtracted from the measured counts. The cell carpets remaining in the incubation flasks after the removal of the reaction mixtures were then digested with 5 mL of 0.1 M NaOH, and their protein contents were determined using the bicinchoninic acid (BCA) assay kit (Thermo Scientific Pierce, Rockford, IL, USA). The rates of total glucose oxidation (pmol glucose/μg protein/60 min) based on the conversion from [^14^C]glucose to ^14^CO_2_ over 60 min were measured.

### Measurement of PPP activity in cultured astroglia and neurons

PPP activity was measured using a modification of the method described by Hothersall et al. [41]. Briefly, cells were incubated with glucose with tracer doses of [1-^14^C]glucose or [6-^14^C]glucose for 60 min, and the PPP rate calculated as the difference between the ^14^CO_2_ derived from [1-^14^C]glucose (metabolized by both PPP and the tricarboxylic acid [TCA] cycle,) and that derived from [6-^14^C]glucose (metabolized by only the TCA cycle) was taken as an indicator of PPP activity.

### Measurement of ROS and NO production

The production of ROS, mainly H_2_O_2_, and NO in cells was assessed by H_2_DCFDA [42] and DAF-FM DA [43] using semiquantitative fluorometric measurement. Immediately prior to the assay, the nutrient medium was removed and the cells were washed twice with PBS without glucose. Then, DBSS containing 10 μmol/L of H_2_DCFDA or DAF-FM DA dissolved in DMSO (final volume of DMSO 0.1 %) supplemented with 2 mmol/L glucose were added, and the cells were incubated at 37 °C in humidified air with 7 % CO_2_ for 30 min. After the H_2_DCFDA or DAF-FM DA were loaded for 30 min, the cells were washed twice again with PBS without glucose and DBSS containing 2 mmol/L d-glucose. The cells were further incubated for 60 min, and the fluorescence level indicating intracellular ROS or NO production was measured at 0 and 60 min using a fluorescent microplate reader (Infinite 200 PRO; Tecan Japan, Kanagawa, Japan) with excitation at 485 nm and emission at 530 nm. As the fluorescent signals linearly increased for up to 60 min (data not shown), the results were expressed as the percent increase in fluorescent signal at 60 min compared with that at 0 min.

### Measurement of total glutathione content using a fluorometric method

The intracellular content of total glutathione in astroglia was assessed using MCB which forms an adduct with glutathione via an enzymatic reaction catalyzed by glutathione transferases [44]. Cells were incubated with MCB (50 μmol/l) for 30 min. The intracellular formation of the GSH–MCB adduct was assessed using a microplate reader with excitation at 360 nm and emission at 465 nm. The results were expressed as the fluorescence signal standardized by cellular protein content.

### Immunohistochemistry

The translocation of Nrf2 from the cytosol to the nucleus was assessed using immunohistochemistry. Astroglial cells grown in a glass-bottomed 35-mm dish were fixed with 4 % paraformaldehyde for 10 min on ice. The cells were permeabilized with 0.5 % Triton X-100 for 10 min at room temperature, followed by post-fixation with 4 % paraformaldehyde for 5 min on ice. For immunostaining, the cells were washed twice with PBS containing MgCl_2_ and CaCl_2_, and nonspecific IgG binding sites were blocked by the incubation of cells in PBS containing 3 % bovine serum albumin and 3 % normal goat serum for 30 min at room temperature. Cells were incubated with primary antibodies (anti-GFAP [1:200], anti-Iba1 [1:200], and anti-Nrf2 polyclonal antibody [1:100]) for 2 h at room temperature. They were then incubated with the secondary antibodies (rhodamine-conjugated goat anti-rabbit IgG antibody or FITC-conjugated goat anti-mouse IgG antibody) with 4′,6-diamino-2-phenylindole (DAPI) for nuclear staining for 1 h. The cells were examined using a laser confocal microscopy system (Leica TCS SP5; Leica Microsystems, Exton, PA, USA).

### Western blotting

Cells were homogenized in an ice-cold lysis buffer; radioimmunoprecipitation assay buffer, 0.5 % sodium dodecyl sulfate, ~0.1 M phenylmethanesulfonyl fluoride, and protease inhibitor cocktail. The concentration of total protein in the supernatant was determined using the BCA protein assay kit (Thermo Scientific Pierce, Rockford, IL, USA). The proteins were separated using 10 % sodium dodecyl sulfate polyacrylamide gel electrophoresis and electrophoretically transferred to polyvinylidene difluoride membranes (Millipore Corporation, Bedford, MA, USA). After treatment for 1 h with a blocking solution containing 5 % nonfat dry milk, the membrane was incubated with anti-TLR4 antibody (1:200), and anti-β actin antibody (1:5000). After washing, the membranes were reacted with the second bodies, the membrane was incubated with horse-radish peroxidase-conjugated anti-goat (Santa Cruz), or anti-mouse antibody (Jackson ImmunoResearch) and the immunoreactive bands were visualized by enhanced chemiluminescence and detected with a lumino-image analyzer (Las-4000; Fujifilm, Tokyo, Japan).

### Evaluation of Nrf2 activation by quantitative reverse transcription PCR (qRT-PCR) analysis for mRNA of heme oxygenase-1 (HO-1)

Total RNA from cultured cells was isolated using RNeasy mini kit (Qiagen, Valencia, CA) with a DNase (Qiagen, Valencia, CA) treatment. First-stranded cDNA was synthesized using PrimeScript RT reagent Kit (TaKaRa) according to the manufacturer’s instructions. qRT-PCR was performed using predesigned gene-specific TaqMan Gene Expression Assays (Applied Biosystems) with the StepOnePlus real-time PCR system (Applied Biosystems). Rat HO-1 TaqMan probe and primers (cat# Rn00561387_m1) and rat GAPDH TaqMan probe and primers (cat# Rn01775763_g1) were from Applied Biosystems. The housekeeping gene glyceraldehyde 3-phosphate dehydrogenase (GAPDH) was used for normalization. The fold difference between levels of transcripts was calculated using the ΔΔCT method.

### Quantification of metabolites by capillary-electrophoresis/electrospray ionization (CE/ESI) MS

The PPP flux and other metabolic pathway of glucose were analyzed using CE/ESI MS (Additional file 1).

### Statistical analyses

Statistical comparisons among the values obtained for each group were performed using grouped *t* tests or one-way analysis of variance followed by the Dunnett test for multiple comparisons. A *P* value of <0.05 was considered statistically significant.

## Results

### Ara-C/LME treatment eliminated microglia in the secondary astroglial culture

**Fig. 1:**
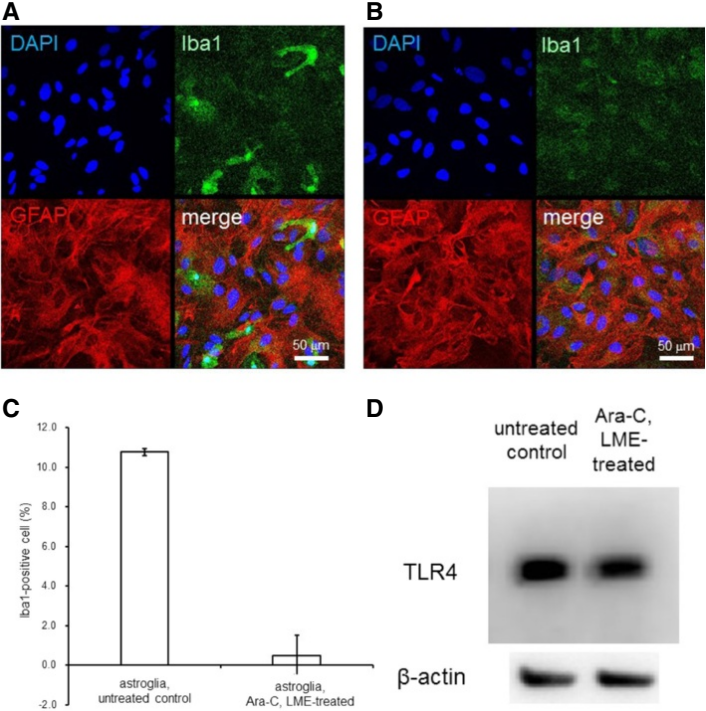
Chemical depletion of microglia by Ara-C/LME. Ara-C/LME treatment removed Iba1-positive cells. Representative photographs showing fluorescent immunostaining of DAPI (*blue*), Iba1 (*green*), or GFAP (*red*) in the secondary astroglial culture. In untreated astroglia, there was a small number of Iba1-positive cells. *Scale bar*, 50 μm (**a**). After Ara-C/LME-treatment, Iba1-positive cells disappeared. Scale bar, 50 μm (**b**). In untreated astroglia, 10.8 ± 0.16 (%) of Iba 1-positive cells were observed, while in Ara-C/LME-treated astroglia, only 0.5 ± 1.0 (%) of cells were Iba1-positive. Values are the means ± SD of four areas (**c**). Western blotting showed that both the normal astroglial culture (with microglia) and Ara-C/LME-treated astroglial culture (without microglia) expressed TLR4 (**d**)

As shown in Fig. 1a, b, the secondary astroglial culture mainly consists of GFAP-positive astrocytes (red). However, immunostaining with Iba1 (green) showed a small number of microglia coexisting with the astroglia (Fig. 1a). After Ara-C/LME-treatment, Iba1-positive cells were completely removed (Fig. 1b). The Iba1-positive cells were counted and shown as % of the total number of cells counted with DAPI (Fig. 1c). The proportion of microglia in the normal astroglial culture was approximately 10 %, as previously reported, but was reduced to zero after treatment with Ara-C/LME. Western blot analysis (Fig. 1d), however, showed TLR4 expression in the microglia-depleted astroglial culture as well as microglia-containing astroglia, indicating that cultured astroglial cells indeed harbor TLR4 and can potentially respond to the LPS stimulation.

### Microglia-enriched culture produced both ROS and NO in response to LPS

**Fig. 2:**
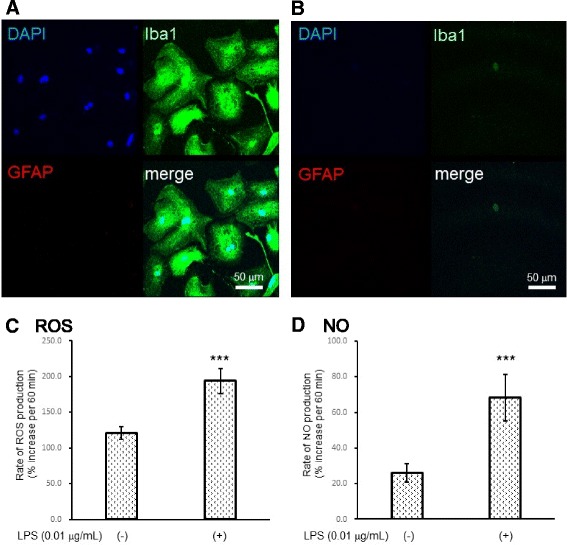
Microglia-enriched culture produced both ROS and NO in response to LPS. Representative photographs showing only Iba1-positive (*green*) cells in the microglial culture. *Scale bar*, 50 μm (**a**). After Ara-C/LME-treatment, Iba1-positive cells disappeared. *Scale bar*, 50 μm (**b**). LPS induced both ROS (**c** *ROS* **)** and NO (**d** *NO*) production. The rate of ROS/NO production was expressed as percent increase in the fluorescent signal at 60 min compared with that at 0 min. Values are the means ± SD of four wells. ***P < 0.001 versus control (grouped *t* test)

We cultured primary microglial cells from primary astroglial cultures. As shown in Fig. 2a, Iba1-positive cells without GFAP-positive cells were obtained. Ara-C/LME treatment eliminated almost all Iba1-positive cells, as expected (Fig. 2b). LPS (0.01 μg/mL for 12 h) induced both ROS and NO production in these Iba1-positive cells (Fig. 2c, d).

### LPS-induced production of ROS and NO disappeared after chemical depletion of microglia

**Fig. 3:**
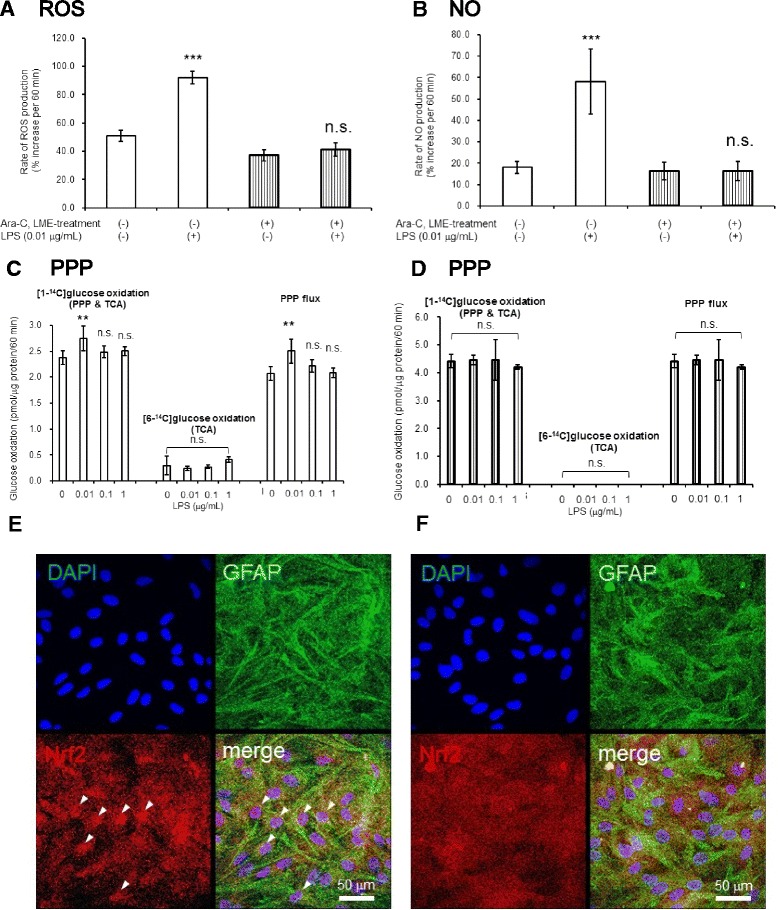
The effects of chemical depletion of microglia. Chemical depletion of microglia eliminated LPS-induced production of ROS and NO and astroglial PPP activation. The rate of ROS/NO production was expressed as percent increase in the fluorescent signal at 60 min compared with that at 0 min. The rate of ROS production in the secondary astroglial culture exposed to LPS (0.01 μg/mL) for 12 h (**a** *ROS*), and the rate of NO production in the secondary astroglial culture exposed to LPS (0.01 μg/mL) for 12 h (**b** *NO*). *Open columns*, untreated astroglia. *Striped columns*, Ara-C/LME-treated astroglia. Values are the means ± SD of six wells. *n.s.*, not significant, ***P < 0.001 versus control (grouped *t* test). PPP flux in untreated astroglia (**c** *PPP*) and in microglia-depleted astroglia (**d** *PPP*) after 15-h exposure to various concentrations of LPS as indicated in the figure. PPP flux increased in untreated astroglia at LPS of 0.01 μg/mL, but not in microglia-depleted astroglia. Values are the means ± SD of four flasks. *n.s.*, not significant, **P < 0.01 versus control (ANOVA followed by Dunnett’s test). Representative photographs showing immunofluorescent staining with DAPI (*blue*), GFAP (*green*), or Nrf2 (*red*) in the secondary astroglial cultures. LPS (0.01 μg/mL) for 12 h induced the nuclear translocation of Nrf2 in microglia-containing astroglia (**e**), but did not in microglia-depleted astroglia (**f**). *Scale bar*, 50 μm

Astroglial cells containing microglia showed marked elevation in the rates of ROS (Fig. 3a) and NO (Fig. 3b) production after exposure to LPS (0.01 μg/mL) for 12 h. However, these increases were eliminated after microglial depletion (Fig. 3a, b).

### LPS induced increases in PPP flux in microglia-containing astroglia, but not in microglia-depleted astroglia

As shown in Fig. 3c, the effect of LPS on increasing PPP flux in astroglia was evident only in the presence of microglia. LPS did not induce PPP activation after the elimination of microglia (Fig. 3d).

### LPS induced nuclear translocation of Nrf2 in astroglia in the presence of microglia

Immunohistochemistry showed that LPS induced Nrf2 translocation into the nuclei of astroglial cells in the presence of microglia (Fig. 3e) but not after microglia depletion (Fig. 3f).

### Inhibition of MAPK eliminated microglial NO production and astroglial PPP activation, indicating that MAPK involvement in LPS-induced PPP activation in astroglia is mediated by microglial NO production

**Fig. 4:**
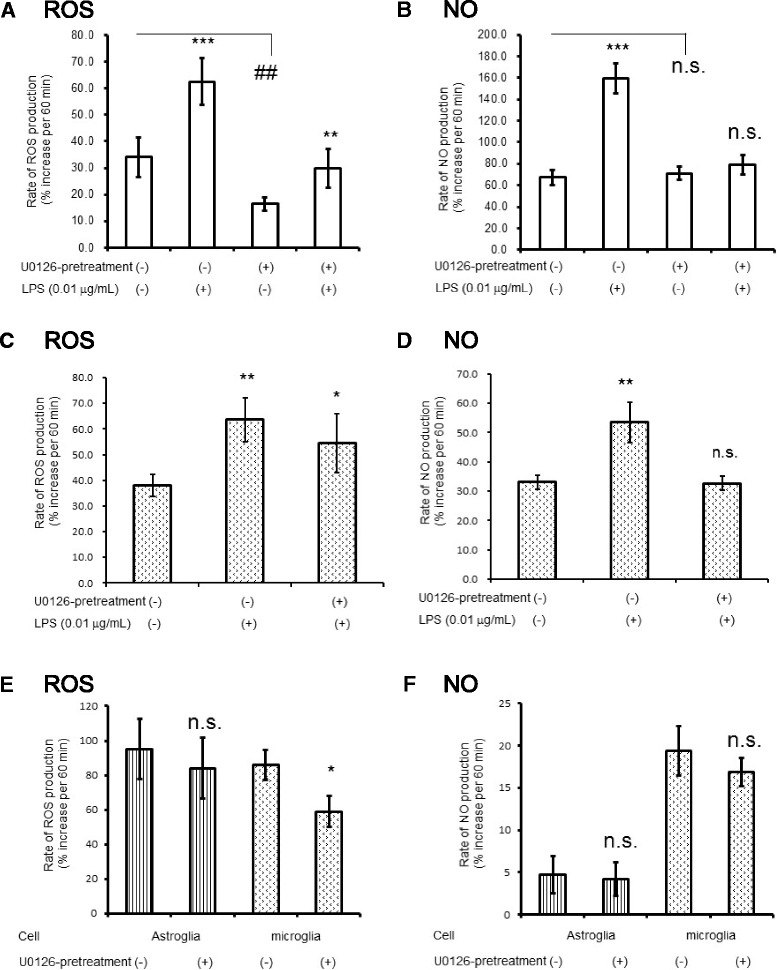
The effects of U0126-pretreatment on microglia and astroglia. U0126 pretreatment eliminated LPS-induced production of NO in untreated astroglia and in microglia. The rate of ROS production (**a** *ROS*) and the rate of NO production (**b** *NO*) in the astroglial culture exposed to LPS (0.01 μg/mL) for 12 h in the presence or absence of U0126 (10 μmol/L). Cells had been incubated with U0126 for 12 h before exposure to LPS. Values are the mean ± SD of six wells. *n.s.*, not significant, **P < 0.01, ***P < 0.001 versus control (grouped *t* test). The rate of ROS production (**c** *ROS*), and the rate of NO production (**d** *NO*) in the microglial culture exposed to LPS (0.01 μg/mL) for 12 h in the presence or absence of U0126 (10 μmol/L). Basal production of ROS and NO in astroglia or microglia was assessed with or without pretreatment of U0126 for 24 h. ROS production was reduced by U0126 in microglia, while astroglial ROS production was not affected (**e** *ROS*). Neither astroglial nor microglial NO production was altered by U0126 pretreatment (**f** *NO*). Values are the mean ± SD of four wells. *n.s.*, not significant, *P < 0.05, **P < 0.01 versus control (grouped *t* test)

As shown in Fig. 4, NO production presumably by microglia in response to LPS was eliminated by U0126, a selective inhibitor of MEK1/2 (Fig. 4b), whereas ROS production induced by LPS was not affected (Fig. 4a). Basal production of ROS, but not NO was reduced by U0126 (Fig. 4a). Similarly, U0126 eliminated NO production by LPS in microglia (Fig. 4d), while ROS production was not affected by U0126 (Fig. 4c). We examined if U0126 treatment affects basal production of ROS or NO in astroglia or microglia. Neither ROS nor NO production in astroglia was affected by U0126, while microglial ROS, but not NO was reduced (Fig. 4e, f).

### NO directly induced Nrf2 activation, leading to PPP activation in astroglia

**Fig. 5:**
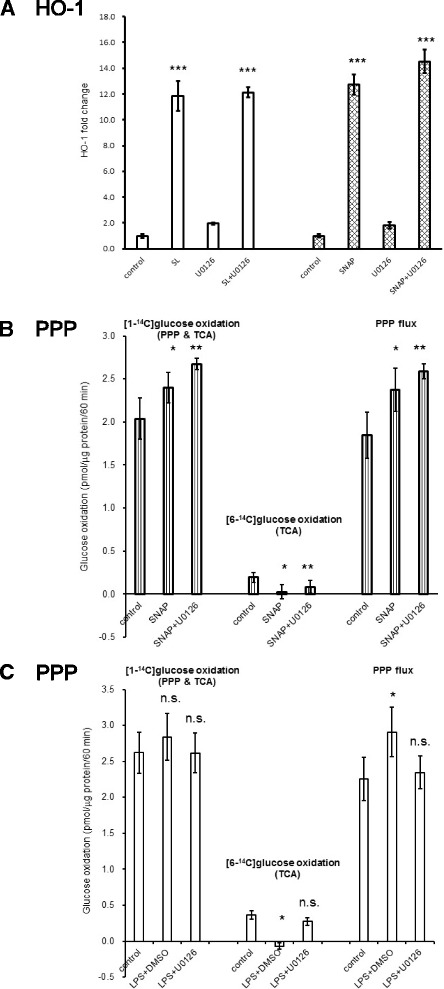
Sulforaphane (*SL*) and NO activate Nrf2 and induce increases in astroglial PPP. Sulforaphane (SL), a well-known activator of Nrf2, increased HO-1 expression and SNAP, an NO donor, also induced HO-1 expression in the presence of U0126 (**a** *HO-1*). mRNA expression of HO-1 in microglia-free astroglia was analyzed by quantitative real-time polymerase chain reaction. HO-1 fold change was calculated relative to each control (means ± SD, *n* = 4). ***P < 0.001 versus each control (grouped *t* test). SNAP induced increases in PPP flux in microglia-depleted astroglia and this NO-induced increases in PPP flux was not eliminated by 12-h pretreatment by U0126 (10 μmol/L), indicating a direct action of NO (**b** *PPP*). However, LPS-induced increases in PPP flux in untreated astroglia were eliminated by 12-h pretreatment by U0126 (10 μmol/L) (**c** *PPP*). Values are the means ± SD of four flasks. *n.s.*, not significant, *P < 0.05, **P < 0.01 versus control (grouped *t* test)

Next, we examined whether NO directly induces the activation of Keap1/Nrf2 system. HO-1 is a representative gene whose transcription is governed by Nrf2. Sulforaphane, a potent Nrf2 activator, was used as a positive control. As shown in Fig. 5a, both sulforaphane and SNAP, an NO donor, induced significant increases in HO-1 gene expression in microglia-free astroglia as assessed by qRT-PCR. Importantly, U0126 did not affect HO-1 gene expression by sulforaphane or SNAP, indicating direct action on Nrf2 activation independent of MAPK. Moreover, NO donor induced PPP activation in astroglia devoid of microglia (Fig. 5b), and these NO-induced PPP activation was not affected by U0126 (Fig. 5b). As shown above, PPP activation in astroglial–microglial culture by LPS was also dependent on the presence of microglia (Fig. 3c, d). We accordingly investigated whether microglia-derived NO or ROS or both induced the activation of astroglial PPP flux. On addition of U0126, which eliminated NO production from microglia (Fig. 4d), the PPP activation in astroglia also disappeared (Fig. 5c), indicating that NO but not ROS is involved in this microglia–astroglia axis.

### Depletion of GSH synthesis induced ROS production in astroglia devoid of microglia in response to LPS

**Fig. 6:**
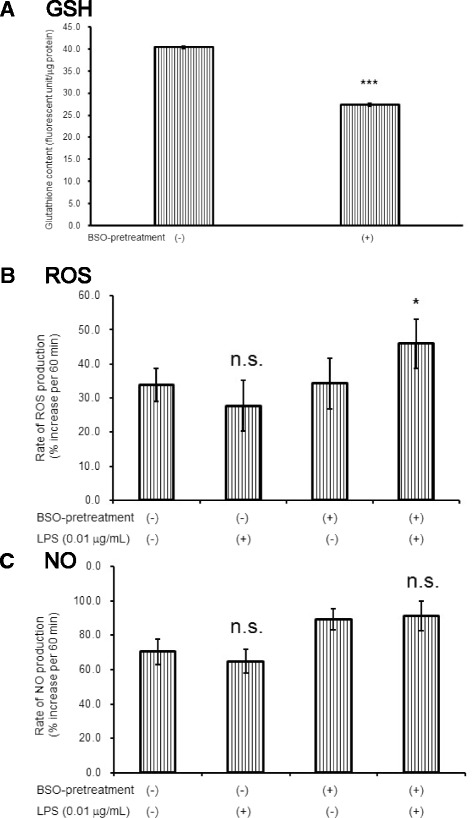
Inhibition of glutathione synthesis uncovers astroglial ROS production. l-buthionine-sulfoximine (BSO; 50 μmol/L for 12 h), an inhibitor of GSH synthesis reduced astroglial GSH content (**a** *GSH*). Values are the means ± SD of four wells. ***P < 0.001 versus control (grouped *t* test). Inhibition of glutathione synthesis in astroglia revealed LPS-induced ROS production in astroglia devoid of microglia. The rate of ROS production in the treated astroglial culture exposed to LPS (0.01 μg/mL) for 12 h (**b** *ROS*), and the rate of NO production in the astroglial culture exposed to LPS (0.01 μg/mL) for 12 h (**c** *NO*) in the presence or absence of BSO. BSO pretreatment (50 μmol/L) had been performed for 12 h prior to exposure to LPS. Values are the means ± SD of six wells. *n.s.*, not significant, *P < 0.05 versus control (grouped *t* test)

As shown in Fig. 1d, astroglia express TLR4. Thus, TLR4-mediated ROS/NO production should be induced in astroglia in response to LPS. We speculated that high astroglial ability to erase ROS masked ROS production. When we inhibited GSH synthesis in astroglia by adding BSO, an inhibitor of GSH synthesis (Fig. 6a), LPS did induce ROS production (Fig. 6b), but not NO production (Fig. 6c) in astroglial cell devoid of microglia.

### Hypoxia enhanced PPP flux in astroglia in both presence and absence of microglia

**Fig. 7:**
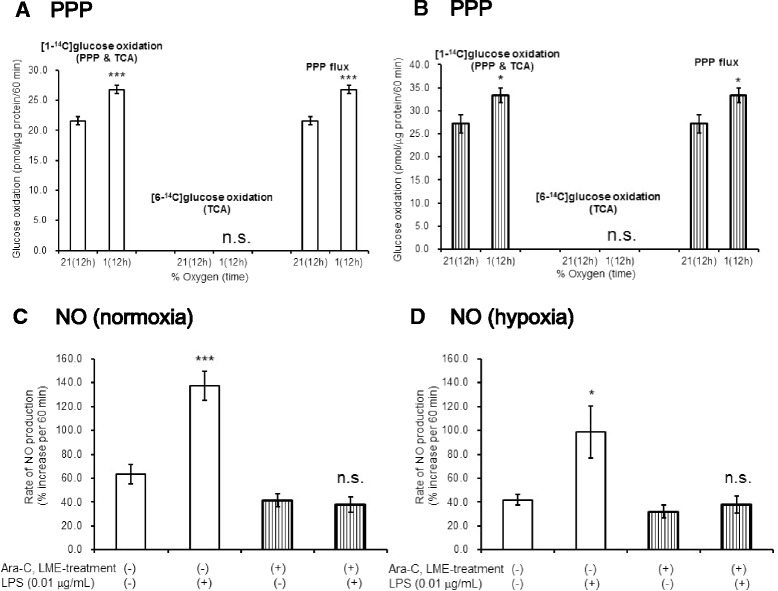
The effects of hypoxia. Hypoxia enhanced PPP flux in microglia-containing and microglia-depleted astroglia, but did not alter NO production. Astroglia containing microglia (**a** *PPP*) and lacking in microglia (**b** *PPP*) had been exposed to 1 % hypoxia for 12 h prior to PPP measurement. Values are means ± SD of four flasks. *P < 0.05, ***P < 0.001 versus normoxia control (grouped *t* test). Astroglia with (*open columns*) or without (*striped columns*) microglia were exposed to 1 % hypoxia and LPS (0.01 μg/mL) prior to NO measurement. Hypoxia did not induce NO production in microglia-depleted astroglia and did not elicit increases in NO production in microglia-containing astroglia (**d** *NO*) as compared with normoxic control (**c** *NO*). Values are mean ± SD of six wells. *P < 0.05, ***P < 0.001 versus control (grouped *t* test)

In contrast to LPS stimulation, hypoxia induced increases in PPP flux in both untreated control astroglia (with microglia; Fig. 7a) and Ara-C/LME-treated astroglia (without microglia; Fig. 7b), indicating that hypoxia-induced PPP activation in astroglia is not dependent on microglia.

### Hypoxia-induced PPP activation in astroglia was not associated with NO production

Hypoxia-induced PPP activation appeared microglia-independent, given that hypoxia enhanced PPP flux in microglia-depleted astroglia. Hypoxia also did not induce NO production in astroglial cells devoid of microglia (Fig. 7d). NO production by microglia-containing astroglia was not enhanced under hypoxia (Fig. 7c, d), indicating that PPP activation under hypoxia was not NO dependent. We speculated that PPP is activated in association with activated glycolysis in response to hypoxia.

### Hypoxia and TLR4 activation by LPS increased astroglial PPP flux in an additive manner

**Fig. 8:**
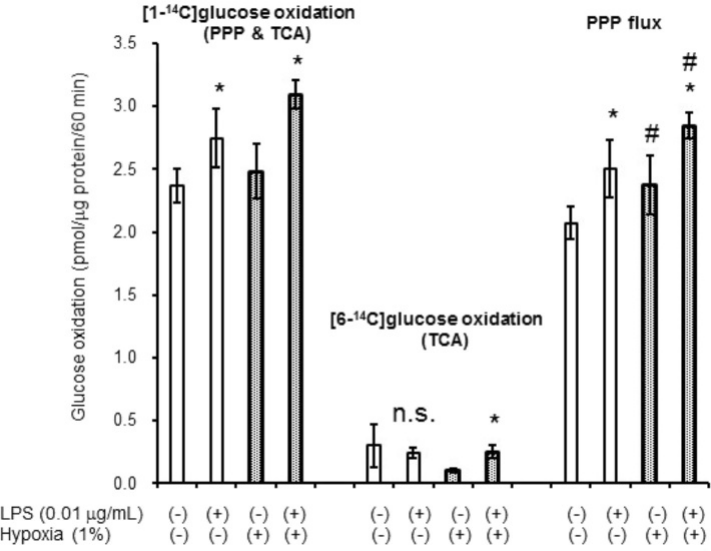
The enhancement of PPP flux by LPS and hypoxia. LPS and hypoxia enhanced PPP flux in astroglia in an additive manner. Microglia-containing astroglia were exposed to LPS (0.01 μg/mL) for 12 h (*open columns*) or LPS and hypoxia for 12 h (*dotted columns*). Values are means ± SD of four flasks. *P < 0.05 compared with LPS(*−*) controls (grouped *t* test). ^#^ *P* < 0.05 compared with normoxia controls (grouped *t* test)

To investigate whether LPS-induced PPP increase is preserved in hypoxic conditions, we assessed the effect of combined hypoxia and LPS on PPP flux. As shown in Fig. 8, hypoxia and LPS enhanced PPP flux in an additive manner, confirming that the two pathways are independent.

### Activation of PPP by sulforaphane, an activator of Nrf2, eliminated LPS-induced ROS and NO production in astroglia containing microglia

**Fig. 9:**
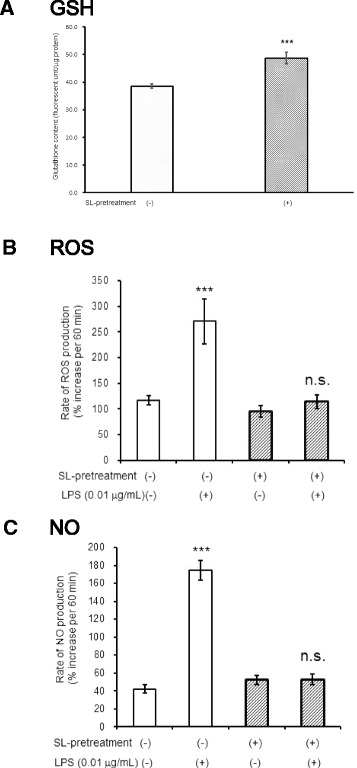
The effects of sulforaphane (SL). SL, a potent activator of Nrf2, increased GSH content (**a** *GSH*) and eliminated LPS-induced increases in ROS and NO. The rate of ROS production (**b** *ROS*) and NO production (**c** *NO*) in untreated microglia-containing astroglia that had been pretreated with DMSO (*open columns*) or sulforaphane (10 μmol/L, *hatched columns*). Values are means ± SD of six wells. ***P < 0.001 versus controls (grouped *t* test)

**Fig. 10:**
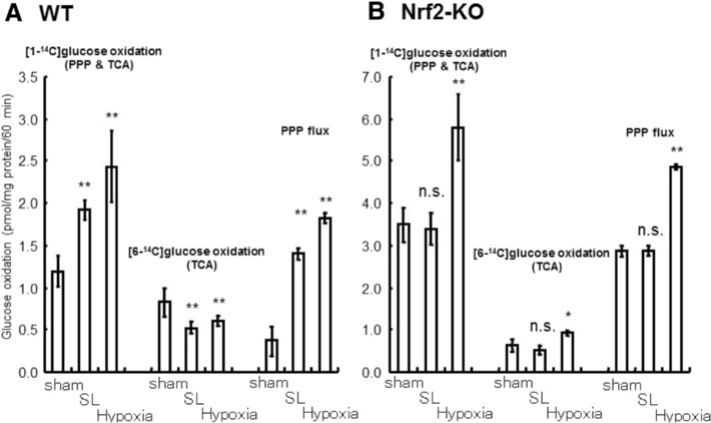
Sulforaphane (SL)- and hypoxia-induced PPP activation in mouse astroglia. Sulforaphane (SL; 10 μM/L) and hypoxia (1 % O_2_ for 12 h) enhanced PPP flux in astroglia prepared from WT (**a** *WT*) and Nrf2-deficient mouse (**b** *Nrf2-KO*). Values are the means ± SD of four flasks. *n.s.*, not significant, *P < 0.05, **P < 0.01 versus control (grouped *t* test)

LPS activates TLR4 in microglia and induces inflammatory responses. In particular, ROS and NO produced by microglia lead to neuronal damage during ischemic stroke. As we have shown, NO derived from microglia enhances PPP activity in astroglia, which may play a role in reducing ROS and NO, thereby protecting neurons. To confirm astroglial protective roles, we measured ROS and NO production in astroglia with microglia after the activation of astroglial PPP. Sulforaphane, a potent activator of Nrf2, activated astroglial PPP and basal ROS production in astroglia [45]. In the present study, we confirmed that sulforaphane induced HO-1 gene expression in astroglia (Fig. 5a). Furthermore, sulforaphane did, indeed, induce increases in glutathione content in astroglia devoid of microglia (Fig. 9a). Consequently, as shown in Fig. 9b, c, LPS-induced robust increases in ROS and NO were completely eliminated. Again, the effects of sulforaphane on PPP activation that was observed in WT mouse (Fig. 10a) were eliminated in astroglia prepared from Nrf2-KO mouse (Fig. 10b). However, hypoxia-induced PPP activation (Fig. 10a) was preserved in astroglia deficient of Nrf2 (Fig. 10b).

### Quantification of metabolites in astroglial pentose-phosphate pathway by capillary-electrophoresis/electrospray ionization (CE/ESI) MS

The metabolites of PPP were increased by hypoxia (1 % O_2_ for 12 h) in astroglia prepared from WT and Nrf2-KO mouse. These results are in accordance with those obtained by the [1-^14^C]/[6-^14^C]glucose method. Namely, the difference of the CO_2_ production from [1-^14^C]glucose and [6-^14^C]glucose is a good indication of the cellular PPP flux. Moreover, hypoxia-induced PPP enhancement was independent of Nrf2 (Additional file 2).

### Neurons do not respond to either LPS stimulation or hypoxia by increasing PPP flux

**Fig. 11:**
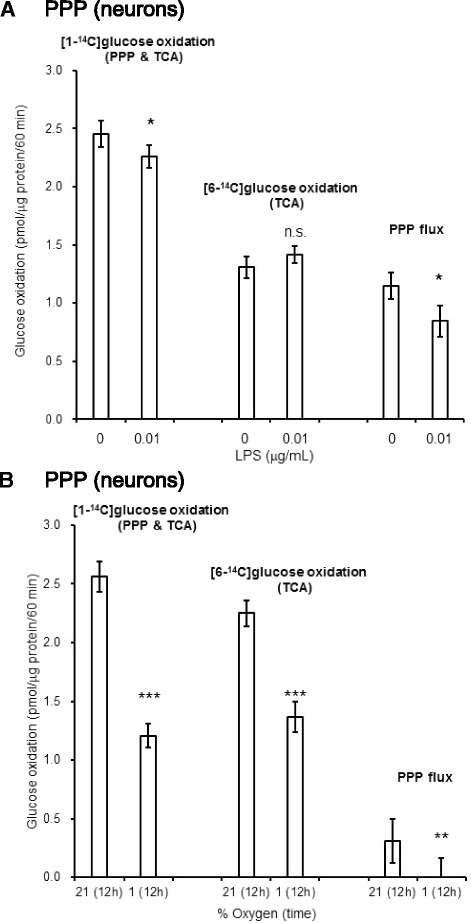
The effects of LPS or hypoxia on PPP flux in neurons. Neither LPS nor hypoxia induce the enhancement of PPP flux in neurons. Neurons were treated with Ara-C when prepared, but not with LME. Both LPS (0.01 μg/mL) (**a** *PPP*) and hypoxia (1 % O_2_) (**b** *PPP*) decreased PPP flux in neurons. Values are means ± SD of four flasks. **P < 0.01 versus normoxia (grouped *t* test). Values are mean ± SD of four flasks. *P < 0.05, **P < 0.01, ***P < 0.001 versus controls (grouped *t* test)

In contrast to astroglia, neurons did not respond to LPS (Fig. 11a) or hypoxia (Fig. 11b) by enhancing PPP flux, indicating that neuronal cells are susceptible to oxidative stress.

## Discussion

Inflammatory responses by neural cells (neurons and glial cells) as well as blood cells have been implicated in the underlying mechanisms of ischemic cell damage [46–53]. TLRs, in particular TLR4, play important roles in the initiation and progression of inflammatory response by intrinsic brain cells, given that brain ischemia induces neuronal release or the increased production of endogenous ligands of TLR4 (damage-associated molecular patterns such as high-mobility group box 1 and peroxiredoxin) in a relatively early stage of an ischemic event, i.e., within 24 h [9, 54, 55]. There is accumulating evidence that not only immune cells in the brain parenchyma, such as microglia, but also astroglia and neurons harbor TLR4 [56–58]. It is likely that microglia respond to these endogenous TLR4 ligands by producing inflammatory cytokines and ROS/NO, leading to further neuronal damage [59]. However, the full role of TLR4 in non-immune cells has not been elucidated. In the present study, we confirmed the presence of TLR4 in astroglia by Western blotting using an astroglial culture devoid of microglia. However, the role of astroglial TLR4 per se does not appear relevant.

The roles of astroglia in ischemic stroke are disputed. Astroglial TLR4 may play either a detrimental or beneficial role or both [60]. Gorina et al. reported that TLR4 stimulation by LPS even at a low concentration (0.01 μg/mL) induced the release of inflammatory cytokines from astroglia, suggestive of a harmful role [13]. For identifying the role of TLR4 in astroglia, cultured cells are useful tools [61–64]. However, usually microglial cells are also present and may affect the results obtained from astroglial cultures [65–68]. In the present study, we obtained a pure astroglial culture using Ara-C/LME-treatment. Although we did not detect cytokine release using this culture, ROS/NO production was not altered by the addition of LPS to the pure astroglial culture, whereas it showed drastic increases in the microglia-containing astroglial culture.

In contrast, Bolaños et al. reported the astroglial activation of PPP flux as a cytoprotective response induced by LPS (1 μg/mL) through NO [16]. PPP is a cytoprotective metabolic pathway of glucose, given that PPP activation induces NADPH production, which is necessary to recycle the oxidized from of GSH back to the reduced form, leading to removal of ROS by GSH peroxidase [69–71]. Importantly, in ischemic stroke, ischemia–reperfusion enhances ROS production in the mitochondria of neurons, leading to further neuronal damage [72, 73]. Thus, PPP activation after ischemia–reperfusion could be an important player in preventing cellular damage during ischemic stroke. Furthermore, they found that NO has a key role in activating PPP in astroglia in response to LPS stimulation [16]. The present study partly supported these beneficial effects of LPS on astroglia. We identified two important findings using two different cultures of astroglia with or without microglia. First, the origin of NO and ROS production in response to LPS stimulation was coexisting microglia. NO elimination by MAPK inhibition eliminated increases in PPP in astroglia, supporting the PPP-activating role of NO. Second, this NO-induced astroglial PPP activation was induced via the Keap1/Nrf2 system in astroglia. Interestingly, MAPK does not seem to be involved in the basal production of NO in microglia. In contrast, basal production of ROS in microglia decreased by MAPK inhibition, indicating different roles of MAPK in microglial production of NO and ROS.

Hypoxia per se induced PPP activation in astroglia by a completely different mechanism. Hypoxia could potentially activate PPP flux, given that the PPP is a branch pathway of glycolysis that is well known to be activated under hypoxic conditions [74, 75]. However, the effects of hypoxia on PPP flux of astroglial cells have been extensively examined [75, 76]. Instead, inflammatory cells such as macrophages alter their metabolic character to increase glycolysis in response to inflammation, which is associated with hypoxic environments. With respect to neurons, oxidative metabolism of glucose is required to produce sufficient ATP to maintain neuronal action potentials [77, 78]. In contrast, astroglia energy metabolism is more dependent on glycolysis [78–80]. We have reported that astroglial glycolysis and its branch pathway, the PPP, are important for protection of neurons against oxygen stress [45]. In the present study, we found that hypoxia-induced activation of the PPP in astroglia is mediated by a different pathway from that mediating NO-induced activation of the PPP and that these two pathways act in an additive manner.

Astroglial cultures always contain a small but appreciable number of microglia [81–83]. The present study showed that a normal astroglial culture contains as much as 10 % of Iba1-positive cells. Elimination of microglia from astroglia is important for distinguishing the astroglial response to TLR4 stimulation. Ara-C combined with LME eliminates microglia effectively [24, 25]. The mechanism by which LME destroys microglia has not been fully established. The lysosomal enzyme, which is abundant in microglia, is involved [29, 30]. In the present study, we confirmed that this treatment succeeded in completely eliminating microglia and that LPS-induced but not hypoxia-induced PPP activation occurred only in the presence of microglia. Interestingly, however, TLR4 expression in pure astroglia devoid of microglia was unaltered, indicating that astroglia per se express TLR4. Thus, LPS stimulation could possibly induce astroglial response through TLR4 activation, whether pro- or anti-inflammatory. We speculated that astroglial PPP activity is high enough to mask ROS production in response to LPS. In fact, LPS did induce increases in ROS production in astroglia in the absence of microglia after GSH depletion. We do not know why LPS did not induce NO production in astroglia.

PPP is a cytoprotective metabolic pathway of glucose, given that PPP activation induces the NADPH production that is necessary to recycle the oxidized form of GSH back to the reduced form, leading to the removal of ROS by GSH peroxidase [84]. The rate-limiting enzyme of PPP, G6PDH, is transcriptionally regulated: the transcriptional factor Nrf2 binds to the ARE located upstream of the G6PDH gene [18, 85]. Nrf2 is released from its adaptor protein Keap1 under stress imposed by ROS and RNS [19]. Thus, ROS or NO can elicit Nrf2 activation, leading to PPP activation. Bolaños et al. indicated that NO induces astroglial PPP activation via mitochondrial inhibition and resulting activation of AMP-activated protein kinase (AMPK) [16]. The results of the present study supported it, since the TCA flux as assessed by [6-^14^C]glucose oxidation decreased by NO. However, increases in PPP flux did not seem to be explained merely by a decreased flux to the TCA cycle. In the present study, we confirmed that NO per se induced Nrf2 activation and increases in PPP flux, and after the inhibition of NO production from microglia by U0126, LPS failed to induce PPP activation, even though ROS production was maintained. NO reportedly activates the Keap1/Nrf2 system by S-nitrosylation of the Keap1 protein, facilitating the release of Nrf2 [21]. ROS may also act as an activator of the Keap1/Nrf2 system through the modification of thiol residues of Keap1 [86–89]. The present study, however, did not support a major role of ROS in activating the Keap1/Nrf2 system.

Another possibility is astroglial TLR4-mediated response via MAPK-associated phosphorylation of Nrf2. The mechanisms by which Nrf2 is activated are multiple and the phosphorylation of Nrf2 at serine 40 is one of them [90–93]. Various kinases may phosphorylate Nrf2 [20]. However, our results showed that nuclear translocation of Nrf2 was induced only when microglia were also present. Thus, the direct involvement of MAPK-induced phosphorylation of Nrf2 in astroglia is unlikely.

TLR4 activation is an important mechanism in ischemic stroke, given that ischemia-induced neuronal damage triggers the release of endogenous ligands for TLR4 [9, 53, 94–96]. In the present study, we also examined the effects of hypoxia per se, given that hypoxia induces marked glycolysis. If glycolysis is activated, PPP, a branch pathway of glycolysis, is simultaneously activated. In fact, PPP in astroglia was activated irrespective of the presence of microglia. LPS and hypoxia showed additive effects on PPP activation in astroglia containing microglia, indicating that hypoxia and TLR4 activation act independently. Hypoxia induced the activation of both glycolysis and PPP. Similarly, LPS induced PPP activation as well as glycolysis. In a recent study, hypoxia induced PPP activation coupled with glycolytic activation [97]. In this context, PPP activation is merely a consequence of glycolytic activation induced by hypoxia. Whether or not LPS-induced activation of glycolysis is dependent on the Nrf2 system is unclear from the results of the present study. These pathways are probably independent, given that LPS-induced increases in PPP flux were preserved under hypoxia. However, in brain ischemia, primary hypoxia causes cellular damage, and endogenous TLR4 ligands are released from the damaged cells. It is reasonable to speculate that hypoxia and TLR4 activation work independently to protect the living body against oxygen stress, given that ROS exerts deleterious effects in the progression of neuronal damage, especially when reperfusion of the occluded artery occurs. The protective mechanism of astroglia may play important roles, and its enhancement may lead to a novel therapeutic strategy for acute ischemic stroke.

Finally, we treated astroglia with sulforaphane to induce Nrf2 activation as evidenced by HO-1 expression. By the pretreatment of astroglia with sulforaphane, which had been confirmed to activate the PPP, ROS and NO production were completely blocked. Thus, the activation of PPP by the enhancement of Nrf2 in astroglia may lead to a novel therapeutic strategy for stroke.

## Conclusions

Astroglia in concert with microglia may play a cytoprotective role to counter oxidative stress in stroke via the activation of PPP. The present study supported the role of NO, which seemed to be derived from microglia upon TLR4 stimulation, in increasing flux in astroglial PPP.

## Additional files

Additional file 1: Quantification of metabolites in astroglial pentose-phosphate pathway by capillary-electrophoresis/electrospray ionization (CE/ESI) MS. (DOCX 20 kb) Additional file 2: The effects of hypoxia on astroglial glucose metabolism of glycolysis, pentose-phosphate pathway, and TCA cycle. (DOCX 212 kb)

## Abbreviations

ANOVA: analysis of variance
Ara-C: cytosine β-d-arabinofuranoside hydrochloride
ARE: antioxidant response element
BCA: bicinchoninic acid
BSO: l-buthionine-sulfoximine
DAF-FM DA: diaminofluorescein-FM diacetate
DAMPs: damage-associated molecular patterns
DAPI: 4′,6-diamino-2-phenylindole
DBSS: Dulbecco’s balanced salt solution
DMEM: Dulbecco’s modified Eagle media
DMSO: dimethyl sulfoxide
ERK1/2: extracellular signal-regulated kinase 1/2
FITC: fluorescein-isothiocyanate
G6PDH: glucose 6-phosphate dehydrogenase
GAPDH: glyceraldehyde 3-phosphate dehydrogenase
GFAP: glial fibrillary acidic protein
GSH: reduced form of glutathione
H_2_DCFDA: 2′,7′-dichlorodihydrofluorescein diacetate
HMGB1: high-mobility group box 1
HIF1α: hypoxia-inducible factor 1α
HO-1: heme oxygenase-1
iNOS: inducible nitric oxide synthase
JAK/STAT: Janus kinases/signal transducers and activators of transcription
Keap1: Kelch-like ECH-associated protein 1
KO: knockout
LPS: lipopolysaccharide
LME: l-leucine methyl ester
MCB: monochlorobimane
MEK1/2: MAPK/ERK kinase1/2
MAPK: mitogen-activated protein kinase
Myd88: myeloid differentiation protein 88
NOX: NADPH oxidase
NO: nitric oxide
Nrf2: nuclear factor-erythroid-2-related factor 2
NFκB: nuclear factor κB
PPP: pentose-phosphate pathway
PBS: phosphate-buffered saline without Ca^2+^ and Mg^2+^
RIPA: radio-immunoprecipitation assay
RNS: reactive nitrogen species
ROS: reactive oxygen species
SDS-PAGE: sodium dodecyl sulfate polyacrylamide gel electrophoresis
SNAP: S-nitroso-*N*-acetyl-dl-penicillamine
Sulforaphane: 1-isothiocyanato-(4R,S)-(methylsulfinyl)butane
TCA: tricarboxylic acid
TLRs: Toll-like receptors
U0126: 1,4-diamino-2,3-dicyano-1,4-bis[2-aminophenylthio] butadiene
WT: wild type

## References

[1] Barone FC, Feuerstein GZ (1999). Inflammatory mediators and stroke: new opportunities for novel therapeutics. J Cereb Blood Flow Metab..

[2] Chamorro A, Hallenbeck J (2006). The harms and benefits of inflammatory and immune responses in vascular disease. Stroke.

[3] Becker KJ (1998). Inflammation and acute stroke. Curr Opin Neurol..

[4] Davies CA, Loddick SA, Stroemer RP, Hunt J, Rothwell NJ (1998). An integrated analysis of the progression of cell responses induced by permanent focal middle cerebral artery occlusion in the rat. Exp Neurol..

[5] Hallenbeck JM (1996). Significance of the inflammatory response in brain ischemia. Acta Neurochir Suppl..

[6] Morioka T, Kalehua AN, Streit WJ (1993). Characterization of microglial reaction after middle cerebral artery occlusion in rat brain. J Comp Neurol..

[7] Zheng Z, Yenari MA (2004). Post-ischemic inflammation: molecular mechanisms and therapeutic implications. Neurol Res..

[8] Szabo C, Ischiropoulos H, Radi R (2007). Peroxynitrite: biochemistry, pathophysiology and development of therapeutics. Nat Rev Drug Discov.

[9] Shichita T, Sakaguchi R, Suzuki M, Yoshimura A (2012). Post-ischemic inflammation in the brain. Front Immunol.

[10] O'Neill LA, Golenbock D, Bowie AG (2013). The history of Toll-like receptors—redefining innate immunity. Nat Rev Immunol.

[11] Okun E, Griffioen KJ, Mattson MP (2011). Toll-like receptor signaling in neural plasticity and disease. Trends Neurosci.

[12] Nicholas SA, Coughlan K, Yasinska I, Lall GS, Gibbs BF, Calzolai L (2011). Dysfunctional mitochondria contain endogenous high-affinity human Toll-like receptor 4 (TLR4) ligands and induce TLR4-mediated inflammatory reactions. Int J Biochem Cell Biol.

[13] Gorina R, Font-Nieves M, Marquez-Kisinousky L, Santalucia T, Planas AM (2011). Astrocyte TLR4 activation induces a proinflammatory environment through the interplay between MyD88-dependent NFkappaB signaling, MAPK, and Jak1/Stat1 pathways. Glia..

[14] Garcia-Nogales P, Almeida A, Fernandez E, Medina JM, Bolanos JP (1999). Induction of glucose-6-phosphate dehydrogenase by lipopolysaccharide contributes to preventing nitric oxide-mediated glutathione depletion in cultured rat astrocytes. J Neurochem..

[15] Ben-Yoseph O, Boxer PA, Ross BD (1994). Oxidative stress in the central nervous system: monitoring the metabolic response using the pentose phosphate pathway. Dev Neurosci..

[16] Bolanos JP, Cidad P, Garcia-Nogales P, Delgado-Esteban M, Fernandez E, Almeida A (2004). Regulation of glucose metabolism by nitrosative stress in neural cells. Mol Aspects Med..

[17] Bergstrom AL, Fog K, Sager TN, Bruun AT, Thirstrup K (2013). Competitive HIF prolyl hydroxylase inhibitors show protection against oxidative stress by a mechanism partially dependent on glycolysis. ISRN Neurosci..

[18] Biswas C, Shah N, Muthu M, La P, Fernando AP, Sengupta S (2014). Nuclear heme oxygenase-1 (HO-1) modulates subcellular distribution and activation of Nrf2, impacting metabolic and anti-oxidant defenses. J Biol Chem..

[19] Kolamunne RT, Dias IH, Vernallis AB, Grant MM, Griffiths HR (2013). Nrf2 activation supports cell survival during hypoxia and hypoxia/reoxygenation in cardiomyoblasts; the roles of reactive oxygen and nitrogen species. Redox Biol..

[20] Niture SK, Khatri R, Jaiswal AK (2014). Regulation of Nrf2-an update. Free Radic Biol Med..

[21] Um HC, Jang JH, Kim DH, Lee C, Surh YJ (2011). Nitric oxide activates Nrf2 through S-nitrosylation of Keap1 in PC12 cells. Nitric Oxide..

[22] Abbas K, Breton J, Planson AG, Bouton C, Bignon J, Seguin C (2011). Nitric oxide activates an Nrf2/sulfiredoxin antioxidant pathway in macrophages. Free Radic Biol Med..

[23] Lee SJ, Lee S (2002). Toll-like receptors and inflammation in the CNS. Curr Drug Targets Inflamm Allergy..

[24] Hamby ME, Uliasz TF, Hewett SJ, Hewett JA (2006). Characterization of an improved procedure for the removal of microglia from confluent monolayers of primary astrocytes. J Neurosci Methods..

[25] Pont-Lezica L, Colasse S, Bessis A (2013). Depletion of microglia from primary cellular cultures. Methods Mol Biol..

[26] Takahashi S, Driscoll BF, Law MJ, Sokoloff L (1995). Role of sodium and potassium ions in regulation of glucose metabolism in cultured astroglia. Proc Natl Acad Sci U S A..

[27] Giulian D, Baker TJ (1986). Characterization of ameboid microglia isolated from developing mammalian brain. J Neurosci..

[28] Saura J (2007). Microglial cells in astroglial cultures: a cautionary note. J Neuroinflammation..

[29] Thiele DL, Kurosaka M, Lipsky PE (1983). Phenotype of the accessory cell necessary for mitogen-stimulated T and B cell responses in human peripheral blood: delineation by its sensitivity to the lysosomotropic agent, L-leucine methyl ester. J Immunol..

[30] Jebelli J, Piers T, Pocock J. Selective depletion of microglia from cerebellar granule cell cultures using L-leucine methyl ester. J Vis Exp. 2015:e52983. doi:10.3791/52983 10.3791/52983 PMC4545156 26275019

[31] Gross J, Ungethum U, Andreeva N, Heldt J, Priem F, Marschhausen G (1996). Glutamate-induced efflux of protein, neuron-specific enolase and lactate dehydrogenase from a mesencephalic cell culture. Eur J Clin Chem Clin Biochem..

[32] Danilov CA, Chandrasekaran K, Racz J, Soane L, Zielke C, Fiskum G (2009). Sulforaphane protects astrocytes against oxidative stress and delayed death caused by oxygen and glucose deprivation. Glia..

[33] Cheung KL, Kong AN (2010). Molecular targets of dietary phenethyl isothiocyanate and sulforaphane for cancer chemoprevention. AAPS J.

[34] Guerrero-Beltran CE, Calderon-Oliver M, Pedraza-Chaverri J, Chirino YI (2012). Protective effect of sulforaphane against oxidative stress: recent advances. Exp Toxicol Pathol..

[35] Singh RJ, Hogg N, Joseph J, Kalyanaraman B (1996). Mechanism of nitric oxide release from S-nitrosothiols. J Biol Chem..

[36] Griffith OW (1982). Mechanism of action, metabolism, and toxicity of buthionine sulfoximine and its higher homologs, potent inhibitors of glutathione synthesis. J Biol Chem..

[37] Gao L, Mejias R, Echevarria M, Lopez-Barneo J (2004). Induction of the glucose-6-phosphate dehydrogenase gene expression by chronic hypoxia in PC12 cells. FEBS Lett..

[38] Amaral AI, Teixeira AP, Martens S, Bernal V, Sousa MF, Alves PM (2010). Metabolic alterations induced by ischemia in primary cultures of astrocytes: merging 13C NMR spectroscopy and metabolic flux analysis. J Neurochem..

[39] Abe T, Takahashi S, Suzuki N (2006). Oxidative metabolism in cultured rat astroglia: effects of reducing the glucose concentration in the culture medium and of D-aspartate or potassium stimulation. J Cereb Blood Flow Metab..

[40] Waniewski RA, Martin DL (2004). Astrocytes and synaptosomes transport and metabolize lactate and acetate differently. Neurochem Res..

[41] Hothersall JS, Baquer N, Greenbaum AL, McLean P (1979). Alternative pathways of glucose utilization in brain. Changes in the pattern of glucose utilization in brain during development and the effect of phenazine methosulfate on the integration of metabolic routes. Arch Biochem Biophys.

[42] Gomes A, Fernandes E, Lima JL (2005). Fluorescence probes used for detection of reactive oxygen species. J Biochem Biophys Methods..

[43] Kojima H, Nakatsubo N, Kikuchi K, Kawahara S, Kirino Y, Nagoshi H (1998). Detection and imaging of nitric oxide with novel fluorescent indicators: diaminofluoresceins. Anal Chem..

[44] Chatterjee S, Noack H, Possel H, Keilhoff G, Wolf G (1999). Glutathione levels in primary glial cultures: monochlorobimane provides evidence of cell type-specific distribution. Glia..

[45] Takahashi S, Izawa Y, Suzuki N. Astroglial pentose phosphate pathway rates in response to high-glucose environments. ASN Neuro. 2012;4. doi:10.1042/an20120002 10.1042/AN20120002 PMC3310304 22300409

[46] Brea D, Sobrino T, Ramos-Cabrer P, Castillo J (2009). Inflammatory and neuroimmunomodulatory changes in acute cerebral ischemia. Cerebrovasc Dis..

[47] Gehrmann J, Banati RB, Wiessner C, Hossmann KA, Kreutzberg GW (1995). Reactive microglia in cerebral ischaemia: an early mediator of tissue damage?. Neuropathol Appl Neurobiol..

[48] Stoll G, Jander S, Schroeter M (1998). Inflammation and glial responses in ischemic brain lesions. Prog Neurobiol..

[49] Wen YD, Zhang HL, Qin ZH (2006). Inflammatory mechanism in ischemic neuronal injury. Neurosci Bull..

[50] Xia W, Han J, Huang G, Ying W (2010). Inflammation in ischaemic brain injury: current advances and future perspectives. Clin Exp Pharmacol Physiol..

[51] Cuenca-Lopez MD, Brea D, Galindo MF, Anton-Martinez D, Sanz MJ, Agulla J (2010). Inflammatory response during ischaemic processes: adhesion molecules and immunomodulation. Rev Neurol.

[52] Denes A, Thornton P, Rothwell NJ, Allan SM (2010). Inflammation and brain injury: acute cerebral ischaemia, peripheral and central inflammation. Brain Behav Immun..

[53] Wang Y, Ge P, Zhu Y (2013). TLR2 and TLR4 in the brain injury caused by cerebral ischemia and reperfusion. Mediators Inflamm..

[54] Hamanaka J, Hara H (2011). Involvement of Toll-like receptors in ischemia-induced neuronal damage. Cent Nerv Syst Agents Med Chem..

[55] Gadani SP, Walsh JT, Lukens JR, Kipnis J (2015). Dealing with danger in the CNS: the response of the immune system to injury. Neuron..

[56] Qiu J, Nishimura M, Wang Y, Sims JR, Qiu S, Savitz SI (2008). Early release of HMGB-1 from neurons after the onset of brain ischemia. J Cereb Blood Flow Metab..

[57] Kuang X, Wang LF, Yu L, Li YJ, Wang YN, He Q (2014). Ligustilide ameliorates neuroinflammation and brain injury in focal cerebral ischemia/reperfusion rats: involvement of inhibition of TLR4/peroxiredoxin 6 signaling. Free Radic Biol Med..

[58] Yoo KY, Yoo DY, Hwang IK, Park JH, Lee CH, Choi JH (2011). Time-course alterations of Toll-like receptor 4 and NF-kappaB p65, and their co-expression in the gerbil hippocampal CA1 region after transient cerebral ischemia. Neurochem Res..

[59] Kacimi R, Giffard RG, Yenari MA (2011). Endotoxin-activated microglia injure brain derived endothelial cells via NF-kappaB, JAK-STAT and JNK stress kinase pathways. J Inflamm (Lond).

[60] Mallard C, Wang X, Hagberg H (2009). The role of Toll-like receptors in perinatal brain injury. Clin Perinatol..

[61] Barbierato M, Facci L, Argentini C, Marinelli C, Skaper SD, Giusti P (2013). Astrocyte-microglia cooperation in the expression of a pro-inflammatory phenotype. CNS Neurol Disord Drug Targets..

[62] Chistyakov DV, Aleshin S, Sergeeva MG, Reiser G (2014). Regulation of peroxisome proliferator-activated receptor beta/delta expression and activity levels by toll-like receptor agonists and MAP kinase inhibitors in rat astrocytes. J Neurochem..

[63] Ko HM, Lee SH, Kim KC, Joo SH, Choi WS, Shin CY (2015). The role of TLR4 and Fyn interaction on lipopolysaccharide-stimulated PAI-1 expression in astrocytes. Mol Neurobiol..

[64] Schafer S, Calas AG, Vergouts M, Hermans E (2012). Immunomodulatory influence of bone marrow-derived mesenchymal stem cells on neuroinflammation in astrocyte cultures. J Neuroimmunol..

[65] Bezzi P, Domercq M, Brambilla L, Galli R, Schols D, De Clercq E (2001). CXCR4-activated astrocyte glutamate release via TNFalpha: amplification by microglia triggers neurotoxicity. Nat Neurosci..

[66] Stellwagen D, Malenka RC (2006). Synaptic scaling mediated by glial TNF-alpha. Nature..

[67] Pascual O, Ben Achour S, Rostaing P, Triller A, Bessis A (2012). Microglia activation triggers astrocyte-mediated modulation of excitatory neurotransmission. Proc Natl Acad Sci U S A..

[68] Saijo K, Winner B, Carson CT, Collier JG, Boyer L, Rosenfeld MG (2009). A Nurr1/CoREST pathway in microglia and astrocytes protects dopaminergic neurons from inflammation-induced death. Cell..

[69] Dodson M, Darley-Usmar V, Zhang J (2013). Cellular metabolic and autophagic pathways: traffic control by redox signaling. Free Radic Biol Med..

[70] Lamonte G, Tang X, Chen JL, Wu J, Ding CK, Keenan MM (2013). Acidosis induces reprogramming of cellular metabolism to mitigate oxidative stress. Cancer Metab..

[71] Morgan WA, Kaler B, Bach PH (1998). The role of reactive oxygen species in adriamycin and menadione-induced glomerular toxicity. Toxicol Lett..

[72] Abramov AY, Scorziello A, Duchen MR (2007). Three distinct mechanisms generate oxygen free radicals in neurons and contribute to cell death during anoxia and reoxygenation. J Neurosci..

[73] Kuroda S, Siesjo BK (1997). Reperfusion damage following focal ischemia: pathophysiology and therapeutic windows. Clin Neurosci..

[74] Marrif H, Juurlink BH (1999). Astrocytes respond to hypoxia by increasing glycolytic capacity. J Neurosci Res..

[75] Cakir T, Alsan S, Saybasili H, Akin A, Ulgen KO (2007). Reconstruction and flux analysis of coupling between metabolic pathways of astrocytes and neurons: application to cerebral hypoxia. Theor Biol Med Model..

[76] Kelleher JA, Chan PH, Chan TY, Gregory GA (1995). Energy metabolism in hypoxic astrocytes: protective mechanism of fructose-1,6-bisphosphate. Neurochem Res..

[77] Wakade AR, Wakade TD (1985). Sympathetic neurons grown in culture generate ATP by glycolysis: correlation between ATP content and [3H]norepinephrine uptake and storage. Brain Res..

[78] Gjedde A, Marrett S, Vafaee M (2002). Oxidative and nonoxidative metabolism of excited neurons and astrocytes. J Cereb Blood Flow Metab..

[79] Hertz L, Peng L (1992). Energy metabolism at the cellular level of the CNS. Can J Physiol Pharmacol..

[80] Hertz L, Peng L, Lai JC (1998). Functional studies in cultured astrocytes. Methods..

[81] Brown DR, Schmidt B, Kretzschmar HA (1996). A neurotoxic prion protein fragment enhances proliferation of microglia but not astrocytes in culture. Glia..

[82] Ciccarelli R, Di Iorio P, D'Alimonte I, Giuliani P, Florio T, Caciagli F (2000). Cultured astrocyte proliferation induced by extracellular guanosine involves endogenous adenosine and is raised by the co-presence of microglia. Glia..

[83] Xiong H, Yamada K, Jourdi H, Kawamura M, Takei N, Han D (1999). Regulation of nerve growth factor release by nitric oxide through cyclic GMP pathway in cortical glial cells. Mol Pharmacol..

[84] Parke DV, Dhami MS, Afzal M (1997). The effect of nutrition on chemical toxicity. Drug Metabol Drug Interact..

[85] Zhang HS, Wang SQ (2007). Nrf2 is involved in the effect of tanshinone IIA on intracellular redox status in human aortic smooth muscle cells. Biochem Pharmacol..

[86] Antelmann H, Helmann JD (2011). Thiol-based redox switches and gene regulation. Antioxid Redox Signal..

[87] Burhans WC, Heintz NH (2009). The cell cycle is a redox cycle: linking phase-specific targets to cell fate. Free Radic Biol Med..

[88] Filomeni G, De Zio D, Cecconi F (2015). Oxidative stress and autophagy: the clash between damage and metabolic needs. Cell Death Differ..

[89] Lu J, Holmgren A (2014). The thioredoxin antioxidant system. Free Radic Biol Med..

[90] Huang HC, Nguyen T, Pickett CB (2002). Phosphorylation of Nrf2 at Ser-40 by protein kinase C regulates antioxidant response element-mediated transcription. J Biol Chem..

[91] Jaiswal AK (2004). Nrf2 signaling in coordinated activation of antioxidant gene expression. Free Radic Biol Med..

[92] Kaspar JW, Niture SK, Jaiswal AK (2009). Nrf2:INrf2 (Keap1) signaling in oxidative stress. Free Radic Biol Med..

[93] Niture SK, Jain AK, Jaiswal AK (2009). Antioxidant-induced modification of INrf2 cysteine 151 and PKC-delta-mediated phosphorylation of Nrf2 serine 40 are both required for stabilization and nuclear translocation of Nrf2 and increased drug resistance. J Cell Sci..

[94] Arumugam TV, Okun E, Tang SC, Thundyil J, Taylor SM, Woodruff TM (2009). Toll-like receptors in ischemia-reperfusion injury. Shock..

[95] Brea D, Blanco M, Ramos-Cabrer P, Moldes O, Arias S, Perez-Mato M (2011). Toll-like receptors 2 and 4 in ischemic stroke: outcome and therapeutic values. J Cereb Blood Flow Metab..

[96] Shichita T, Ago T, Kamouchi M, Kitazono T, Yoshimura A, Ooboshi H (2012). Novel therapeutic strategies targeting innate immune responses and early inflammation after stroke. J Neurochem..

[97] Brekke EM, Morken TS, Wideroe M, Haberg AK, Brubakk AM, Sonnewald U (2014). The pentose phosphate pathway and pyruvate carboxylation after neonatal hypoxic-ischemic brain injury. J Cereb Blood Flow Metab..

