# Supplementary material for: A possible role of microglia-derived nitric oxide by lipopolysaccharide in activation of astroglial pentose-phosphate pathway via the Keap1/Nrf2 system
Source: J Neuroinflammation. 2016 May 4;13:99. doi: 10.1186/s12974-016-0564-0 (PMC4855896; doi:10.1186/s12974-016-0564-0)
Supplement: Additional file 1: — Quantification of metabolites in astroglial pentose-phosphate pathway by capillary-electrophoresis/electrospray ionization (CE/ESI) MS. (DOCX 20 kb) [file 12974_2016_564_MOESM1_ESM.docx]

**Quantification of metabolites by capillary-electrophoresis/electrospray ionization (CE/ESI) MS**

Quantitative metabolome analysis method was carried out using capillary electrophoresis mass spectrometry [1]. Briefly, to extract the metabolites, astroglia that had been prepared from wild-type (WT) and Nrf2 gene knockout (KO) mice and cultured in petri dishes (Sumitomo Bakelite, Tokyo, Japan) were exposed to normoxia (21%) or hypoxia (1%) for 12 h. When confluent cells were exposed to hypoxia, cells were placed in a hypoxia chamber (Bugbox Hypoxic Workstation, model INVIVO2 300, RUSKINN, Ltd. Central science trade, TOKYO) preset to 37°C and 1% O_2_. Then, cells were washed in 10% mannitol (Wako) and then plunged into ice-cold methanol to inactivate enzymes. Such a process was conducted in the O_2_-controlled glove box to maintain the proper oxygen tensions. After a 5-min incubation, chloroform and ultrapure water (LC/MS grade; Wako) were added to the solution. The suspension was then centrifuged at 15,000 ×g for 15 min at 4°C. After centrifugation, the aqueous phase was ultra-filtered through a Millipore 5-kDa-cutoff filter to remove proteins. The filtrate was concentrated with a vacuum concentrator (SpeedVac; Thermo, Yokohama, Japan); this condensation process helps quantitate trace levels of metabolites. The concentrated filtrate was dissolved in 50 µL of ultrapure water and used for CE-MS.

All CE/ESI/MS experiments are performed using an Agilent CE Capillary Electrophoresis System equipped with an air pressure pump, an Agilent 1200 series MSD® mass spectrometer and an Agilent 1200 series isocratic high performance liquid chromatography pump, a G1603A Agilent CE/MS adapter kit, and a G1607A Agilent CE/MS sprayer kit (Agilent Technologies). System control, data acquisition, and MSD® data evaluation are performed using G2201AA Agilent ChemStation software for CE-MSD®.

We used internal (added to tissue before extraction) and external (added to sample after extraction) standard compounds. To normalize the shifts of migration (retention) times among datasets, we used two internal standards compounds set to be eluted at the first and the last to measure the time difference between two standard peaks as a calibrant. We then corrected the relative migration times of all peaks by normalizing those of internal standards. For anionic metabolites, we used 2-morholinoethanesulfonic acid (MES) and 1,3,5-benzene-tricarboxylic acid (trimesate). For cationic metabolites, we used L-methionine sulfone and 3-aminopoyrrolidine dihydrochloride. These compounds are not present in the tissues; thus, serving as ideal standards. Loss of metabolites during sample preparation was also corrected. Furthermore, before sample measurements, we measured the mixture of authentic compounds of target metabolites at three different concentrations to generate calibration curves. Quantification (amounts of metabolites in the unit of nmol/mg protein) was done by comparing their normalized peak areas against a calibration curves.

Isomeric species, such as glucose 1-phosphate, glucose 6-phosphate, and flucose 6-phosphate, can be hard to distinguish by MS/MS per se. However, we took advantage of the CE/ESI/MS system [1] where these species are eluted at the different retention time by capillary electrophoresis due to their differential mobility and/or chemical properties. The system is effective in separation of isobaric or isomeric compounds.

**Reference**

1. Soga T, Ohashi Y, Ueno Y, Naraoka H, Tomita M, Nishioka T. Quantitative metabolome analysis using capillary electrophoresis mass spectrometry. J Proteome Res. 2003;2:488-94.
