# Supplementary material for: A possible role of microglia-derived nitric oxide by lipopolysaccharide in activation of astroglial pentose-phosphate pathway via the Keap1/Nrf2 system
Source: J Neuroinflammation. 2016 May 4;13:99. doi: 10.1186/s12974-016-0564-0 (PMC4855896; doi:10.1186/s12974-016-0564-0)
Supplement: Additional file 2: — The effects of hypoxia on astroglial glucose metabolism of glycolysis, pentose-phosphate pathway, and TCA cycle. (DOCX 212 kb) [file 12974_2016_564_MOESM2_ESM.docx]

**Supplemental Figure 1 (Figure S1) 　　The effects of hypoxia on astroglial glucose metabolism of glycolysis, pentose-phosphate pathway, and TCA cycle.**

**A.**

**B. Σ Glycolysis C. Σ TCA cycle D. Σ PPP**

**(A)** The astroglial cells prepared from wild-type (WT) or Nrf2 gene knockout (KO) mice were treated with normoxic (21% O_2_) or hypoxic (1% O_2_) conditions for 12 h. Data show amounts of metabolites in glycolysis, pentose-phosphate pathway (PPP) and TCA cycle. G1P, glucose 1-phosphate; G6P, glucose 6-phosphate; F6P, fructose-6-phosphate; F1,6P, fructose 1,6-bisphosphate; GA3P, glyceraldehyde 3-phosphate; DHAP, dihydroxyacetone phosphate; 3PG, 3-phosphoglycerate; PEP, phosphoenolpyruvate; 6PG, 6-phosphogluconate; Ru5P, ribulose-5-phosphate; R5P, ribose-5-phosphate; Xu5P, xylulose-5-phosphate; S7P, sedoheptulose-7-phosphate; E4P, erythrose-4-phosphate. **(B)** Σ Glycolysis indicates total sum values of G1P, G6P, F6P, F1,6P, GA3P, DHAP, 3PG and PEP. **(C)** Σ TCA cycle indicates total sum values of citrate, cis-aconitate, 2-oxoglutarate, succinate, fumarate, and malate. **(D)** Σ PPP indicates total sum values of 6PG, R5P, Ru5P and S7P. Data indicate the mean ± SE (μmol/g protein) of quadruplicate dishes. **P<0.01 and *P<0.05 versus normoxia (ANOVA with Fischer’s multiple comparison test).
